# Prevalence and dental effects of infant oral mutilation or *Ebiino* among 3–5 year–old children from a rural district in Uganda

**DOI:** 10.1186/s12903-019-0890-6

**Published:** 2019-09-02

**Authors:** Norman Musinguzi, Arthur Kemoli, Isaac Okullo

**Affiliations:** 1 Department of Dentistry, School of Health Sciences, Makerere University, Kampala, Uganda; 2 Department of Paediatric Dentistry and Orthodontics, School of Dental Sciences, University of Nairobi, P.O. Box 34848, Nairobi, 00100 Kenya

**Keywords:** *Ebiino*, Infant Oral mutation, Primary canine, Uganda

## Abstract

**Background:**

*Ebiino*, a form of Infant Oral Mutilation (IOM), involves the gauging or enucleation of primary canine tooth buds in infants, and is believed to be a form of remedy to a range of childhood diseases. The effects of this practice have ranged from the child experiencing excessive bleeding, opportunistic infections and even death, besides the potential negative dental effects on primary and the developing permanent dentition of the affected child. The purpose of the study was to establish the occurrence of *Ebiino* and its dental effects in a rural child-population in Uganda.

**Methods:**

This study formed part of a larger descriptive cross-sectional study on dental caries and gingivitis, in which 432 children aged 3–5 years old from Nyakagyeme Sub-county, Rukungiri District, Uganda, participated.

All the 432 participants (230 males and 202 females, mean age 4.1 *SD* = 0.8) who had been recruited through stratified random sampling procedure, and whose caregivers had provided a written informed consent, were included in the study. Initially the past dental history of each participant was obtained, and all the children had an oral examination carried out to establish their dental status.

**Results:**

The data gathered were entered in a computer and analysed using Windows SPSS version 23.0. The results of the analysis showed the prevalence of missing teeth not due to reasons like caries or trauma was 8.1%, with the primary canine being the most commonly missing tooth. These unusual missing teeth were attributed to a traditional practice called *Ebiino*. Chi-square test showed no statistically significant association of *Ebiino* with gender and age (*p* = 0.352 and *p* = 0.909, respectively). Also found in the study were enamel hypoplasia or damage of some primary canines and/or the primary lateral incisors and first primary molars, as well as displacement of adjacent teeth, a result found to be associated with the practice.

**Conclusion:**

The practice of *Ebiino* appears to be endemic within the communities in Rukungiri region in spite of the negative impacts in form of hypoplasia, midline shift, trauma, dental displacement and missing adjacent teeth that it had on the primary dentition of the child.

## Background

“*Ebiino*” is a term used in the Runyakole-Rukiga dialect from South-western Uganda to mean “false teeth”, but it is a form of Infant Oral Mutilation (IOM) practiced in the community. This practice is also known in other Ugandan communities by different names depending on the region and the native language spoken [1, 2]. It is referred to as *Ebinyo* by Baganda, *Bino* by the Basoga, *Ikela/Icela* by the Itesots of the Eastern Region of Uganda, *Gidog* by the Langi from Northern Uganda, *Lake jo marak* by Japadhola and *Two lak* by Acholi [2]. Other terms have also been used in literature by different authors to describe the same practice: *nylon teeth*, *false teeth*, *primary canine tooth bud enucleation*, *germectomy, tooth bud gouging*, etc. [3].

*Ebiino* or IOM has mostly been practiced in less developed communities in Africa, particularly, in those communities of lower literacy levels and low socio-economic status [1–4]. However, some of those considered educated within the communities, sometimes practice *Ebiino* to their children as a tradition. Further, as a result of human migration, *Ebiino* can even be found in children living in developed nations too [5, 6].

The practice of *Ebiino* involves rubbing of the gums above the developing primary canine tooth bud area with herbs by a traditional healer prior to the gauging out of the tooth buds [1, 2, 4]. It is usually performed when the child is between 4 and 8 months of age but can still be carried out in children up to the age of 18 months. The gum swelling in the canine area is considered to be worms and hence the need to remove them [2, 4, 7]. This timing coincides with the period of the transitioning of the child’s immunity from maternal immunity acquired at birth to humoral immunity. During this period the child is more susceptible to childhood infections and fevers [4]. The soft, not fully mineralised tooth buds are often enucleated by an elderly person or traditional healer in the community using crude, unsterilized basic sharp tools such as hooks and knives on the premise that the swollen blanched areas of the canine region are related to the cause of the childhood illness. It is hoped that the removal of what is usually described as “worms” or “maggots”, would prevent or treat these childhood diseases and prevent death [1–5, 8].

The consequences reported for this practice have included infections and even fatalities of the affected children, missing adjacent primary and permanent teeth (especially the canines), enamel hypoplasia of the permanent canines and/or adjacent teeth, malformation of permanent canines, malocclusions and psychological or social embarrassment [2, 3]. Yet, there are no known benefits arising from this traditional practice, other than just beliefs by the community that it cures/prevent childhood illnesses.

The purpose of the present study was to determine the occurrence of *Ebiino* and its effects on dentition in a rural child population in Uganda.

## Methods

This study, which formed part of a larger descriptive cross-sectional survey on the prevalence and treatment needs of Dental caries and Gingivitis among 3–5-year old children, was conducted in October 2016, in Nyakagyeme sub-county, Rukungiri district, South-western Uganda. The sub-county, which is purely a rural community with subsistence farming as the main economic activity, has 8 divisions called parishes. In the last Uganda national population census of 2014, the sub-county had an estimated child-population of about 6000 children aged five years and below.

Ethical clearance for the study was sought and obtained from Kenyatta National Hospital-University of Nairobi Ethics and Research Committee (Ref: P460/06/2016) and the School of Health Sciences Institution Review Board and Ethics Committee, Makerere University, Kampala, Uganda (SHSREC REF: 2016–036). Written informed consent was obtained from the children’s parents/guardians with assent obtained from the participating children. Any sick, uncooperative child and a child whose caregiver had not provided consent to participate in the study, was excluded from the study. The larger study on the “prevalence and treatment needs of Dental caries and Gingivitis among 3-5-year old children in Nyakagyeme sub-county”, involved a study population drawn from 25 primary schools in Nyakagyeme Sub-county with pre-primary/nursery sections.

**Fig. 1:**
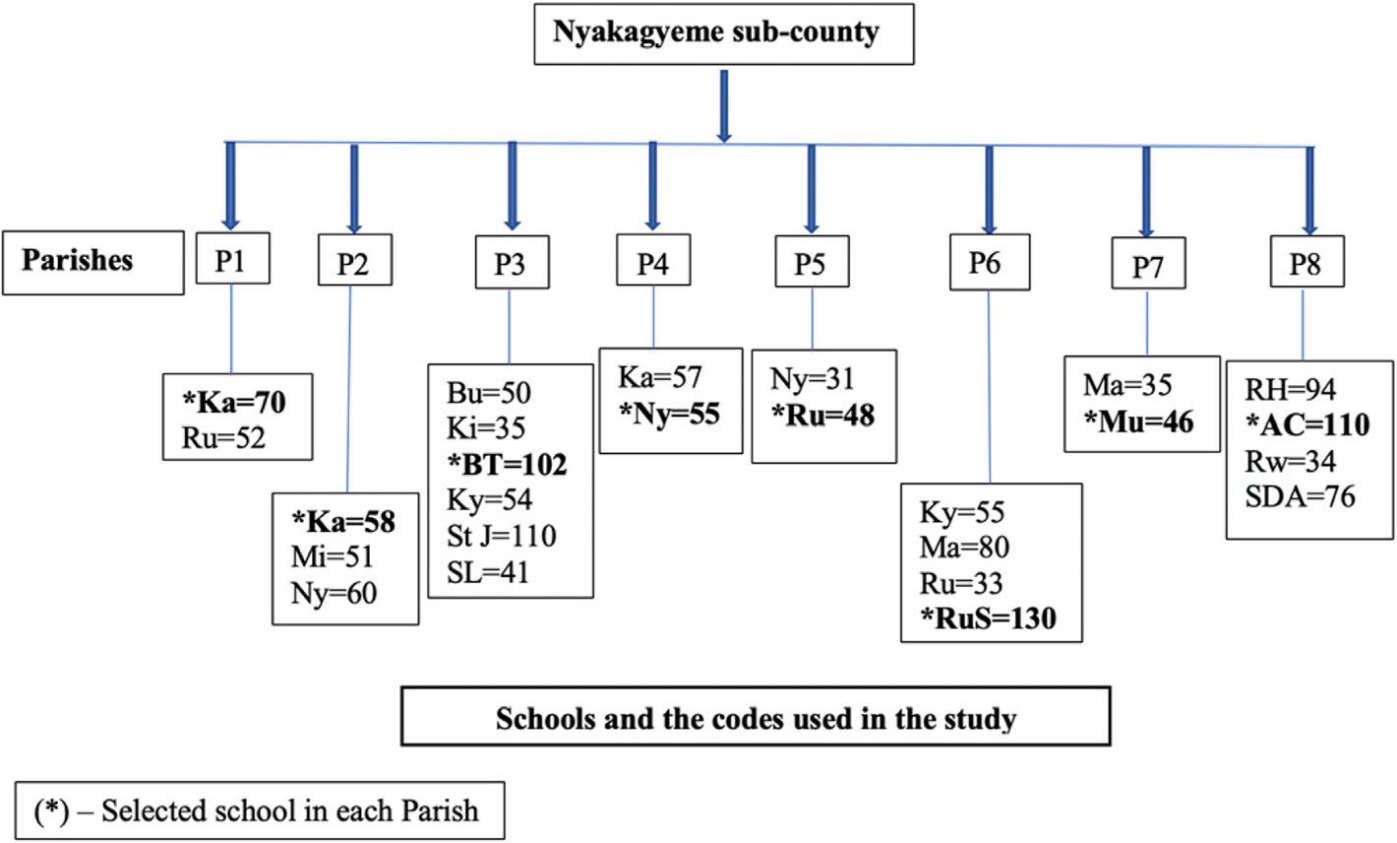
Sampling method applied when selecting the schools that participated in the study

Using stratified random sampling procedure to select the schools in the study, and with the aim of selecting at least a school in each of the 8 parishes in the sub-county, 8 strata were formed. The parishes were coded from P1 to P8, and a list of the schools in each parish was generated and assigned a unique code. The number of pupils attending nursery in each school was also obtained and schools with at least 50 pupils or more (18 schools) were eligible for selection. Ballots with the number assigned to each school were made and dropped in a box for each parish. In case a parish had no school with at least 50 pupils in nursery, the school, with the highest number of pupils was selected for inclusion in the study. The Principle Investigator (PI) then randomly selected one school from each parish by picking a ballot from the box, which indicated the chosen school that formed one of the 8 schools selected in the Sub-county. The total number of children eligible for participation in the study from the selected schools was 619 children as shown in Fig. 1.

The school register in each school was then used as the sampling frame to select the children to participate in the study provided they met the inclusion criteria for participation in the survey - parents had provided written informed consent for their participation in the study and assent from the child to be examined. The children were examined for *Ebiino*, besides other examinations that had been prescribed in the larger study.

The study population for the larger study had been calculated with assumption that children below 6 years in the sub-county was below 10,000 and the prevalence of caries among them to average 50%, a 95% confidence level being considered and 5% degree of accuracy. This gave the number of participants to be 384. However, a total of 432 children in the sub-county aged 3 to 5 years (230 males and 202 females; mean age 4.1, *SD* = 0.8) met the inclusion criteria and were examined during the study. The individual results of the examination that included the *Ebiino* and the other aspects of the larger study, were recorded using a modified Form adopted from the recommended WHO format for assessing oral health for children below 6 years. The age and gender of the participating children were also documented.

The Principle Investigator carried out an intra-oral examination under a natural light source, using a dental mirror, periodontal probe and disposable gloves. The PI examined for missing primary canines and any other primary tooth whose absence could not be attributed to tooth loss resulting from decay or trauma. A missing tooth was scored for dental caries or trauma if a history of early extraction due to caries or trauma had been proffered and confirmed, and in the case of caries that it could also be explained by the decay pattern on the contra-lateral quadrant(s) of the child. Any other soft and hard tissue features associated with these missing teeth were also recorded including the condition of the adjacent teeth. The data gathered were properly coded and entered in the statistical package for social research (SPSS Inc. Version 23.0 for Windows, Illinois, Chicago, USA) cleaned and analysed. Chi-square test was used to compare and relate the variables, with the *P* < 0.05 considered statistically significant. The confidence interval was set at 95%.

## Results

Of the 432 children examined during the study, 48 (11.1%) of them had a missing tooth/teeth. The children who had teeth extracted as a result of dental caries were 13 (3%). There were teeth noted to be missing for other reasons not related to dental caries, trauma or any other pathology. Of the 35 (8.1%) children found with missing teeth attributed to the traditional practice, 16 (3.7%) and 19 (4.4%) were male and female participants respectively. The difference in occurrence of these missing teeth due to other reason between males and females was not statistically significant. The age distribution of the missing teeth due to other reasons was 10 (2.31%), 11 (2.55%) and 14 (3.24%) for the 3-, 4- and 5-year-olds respectively. The difference in the distribution of the missing teeth due to other reasons with age was also not statistically significant, (see Table 1).

**Table 1:** Distribution of missing teeth due to other reasons by gender and age of the participants in the study

Overall	Missing teeth due to other reasons n (rate %) 35 (8.1%)	Chi Value	*p*-value
Gender			
Male	16 (3.7%)	X^2^ = 0.8666	0.352
Female	19 (4.4%)		
Age			
3 Years	10 (2.31%)	X^2^ = 0.1911	0.909
4 Years	11 (2.55%)		
5 Years	14 (3.24%)		

**Fig. 2:**
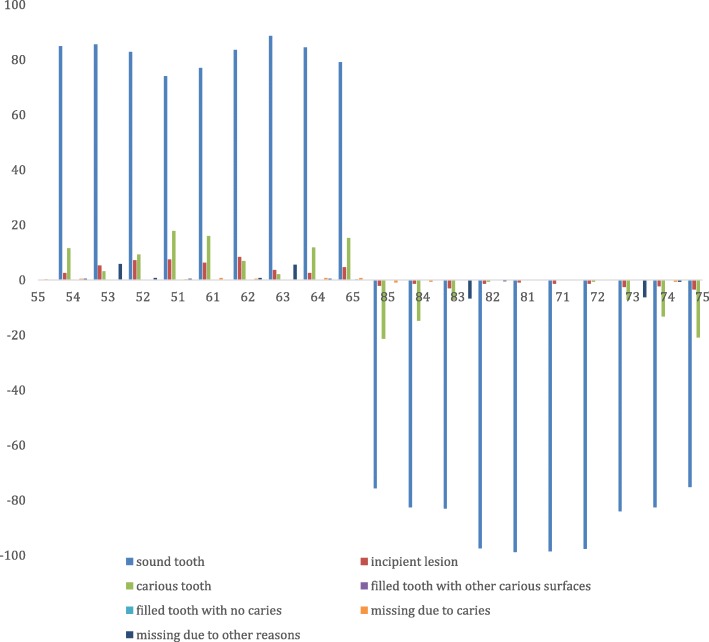
Distribution of the dental findings per tooth in the study participants

**Fig. 3:**
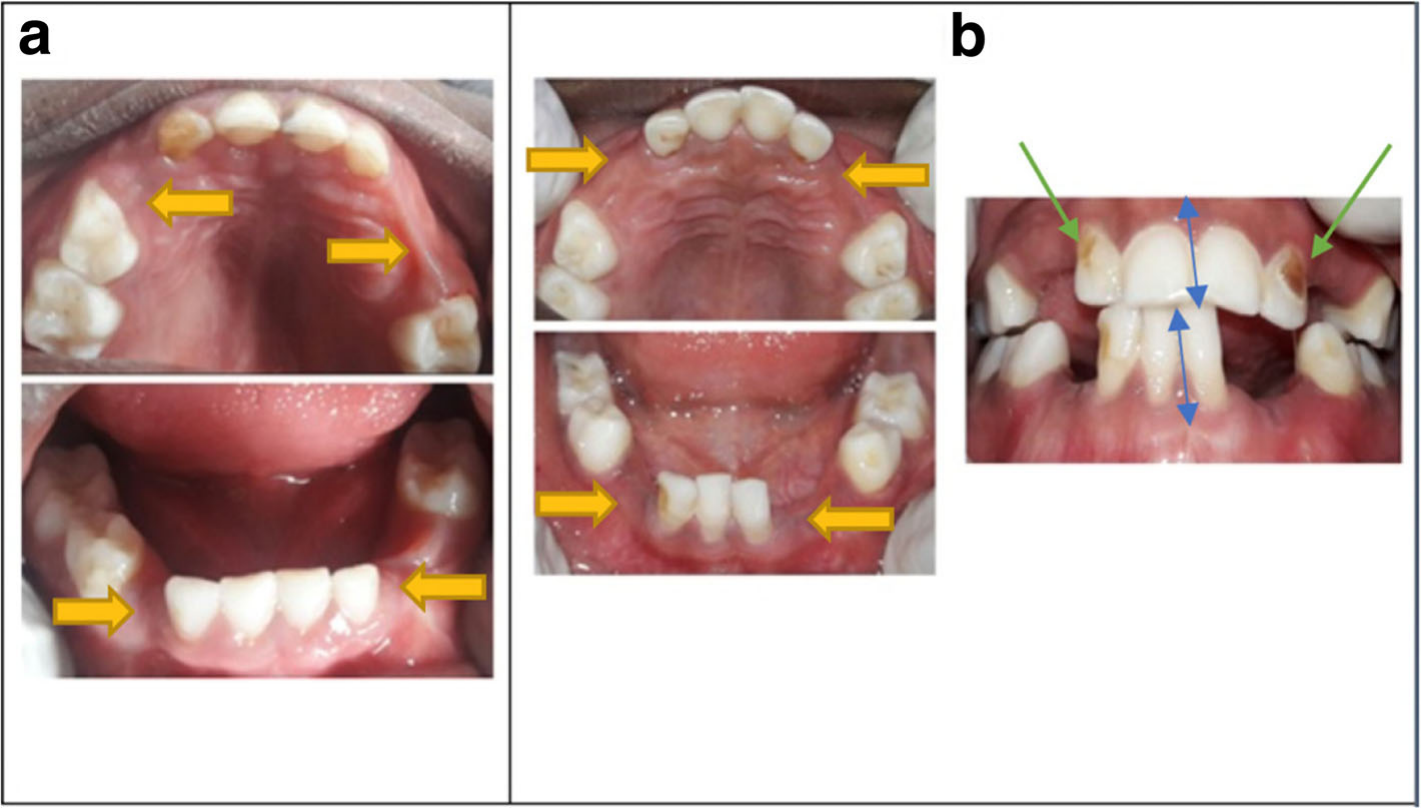
The dental effects of *Ebiino* as observed among the study participants (see **a** & **b**) - missing primary canines, lateral incisors, first molars (yellow arrows), hypoplasia to lateral incisors (green arrows) displacement and shifting of teeth (blue arrows)

**Fig. 4:**
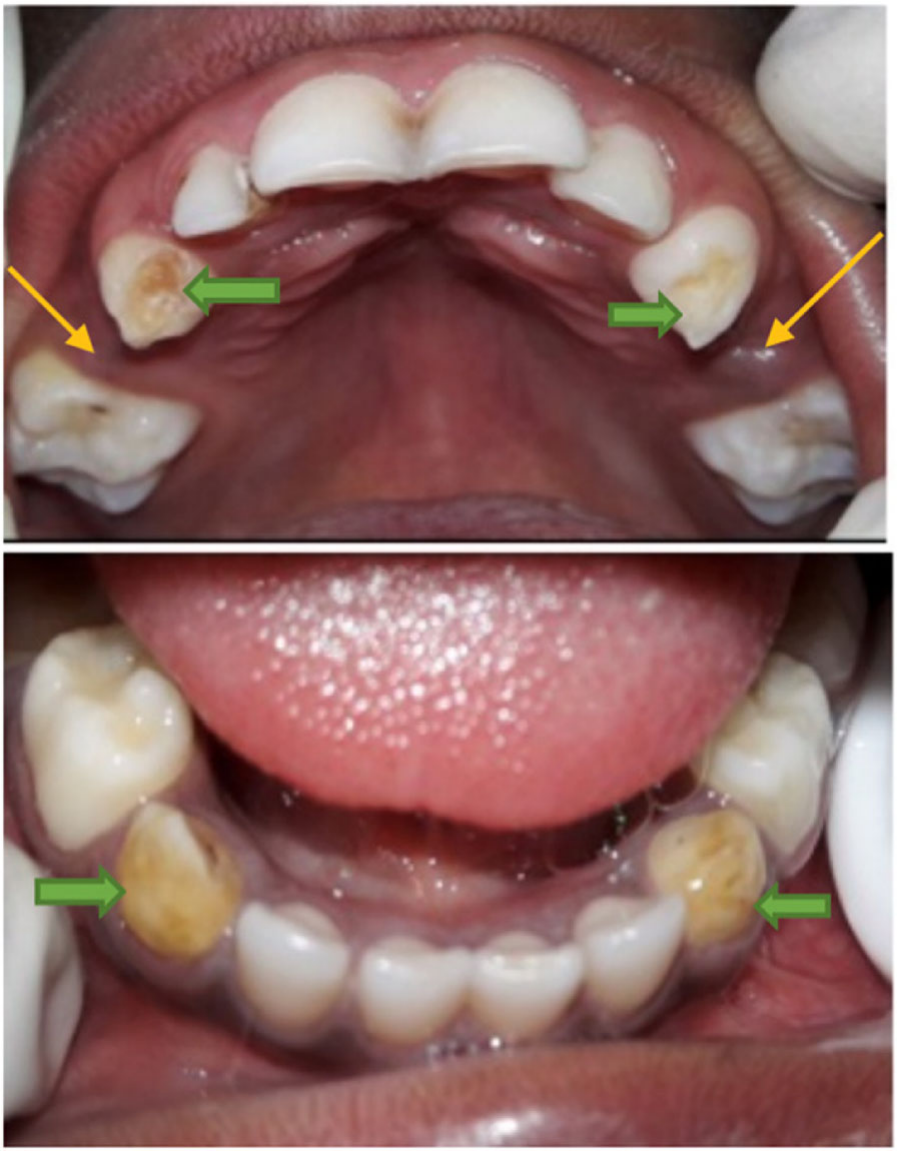
The collateral damage to teeth (see arrows) during the process of undertaking IOM in one of the participants in the study

**Fig. 5:**
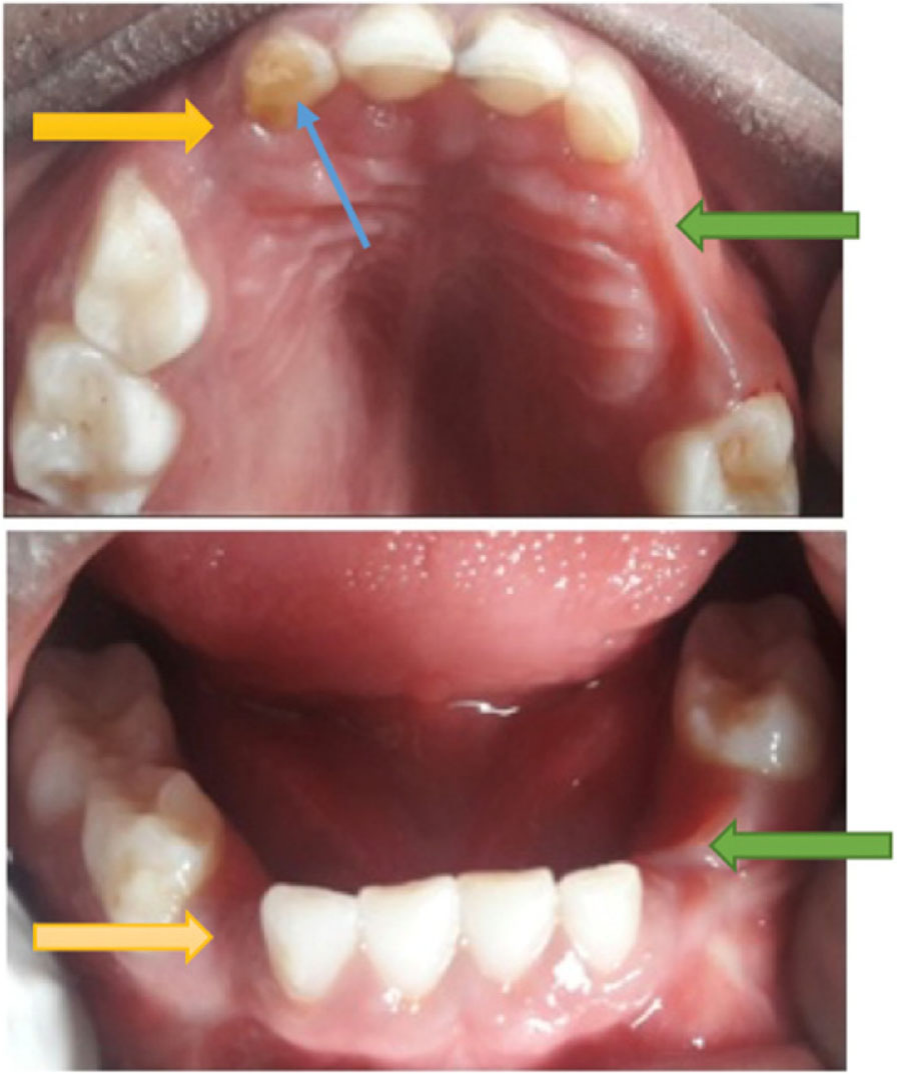
A participant in the study with collateral damage during IOM practice and which included missing primary first molars and canines on the left (green arrows), and only missing primary canines on the right (yellow arrows) and hypoplasia to the primary lateral incisor (blue arrow)

The missing teeth due to other reasons was found to be the primary canines both in the maxilla and mandible, and to a lesser extent, the primary lateral incisors and/or first primary molars (Table 2 and Fig. 2) were also found missing. The frequency of a primary canine missing per quadrant among the study participants was 0.3%. Associated with the missing primary canines, were also conditions like, hypoplasia and/or damage to the crown of adjacent tooth (6 children) or a primary canine (2 children) (see Figs. 3, 4 and 5). One (1) child was found to have a lower midline shift as a result of displacement of the remaining primary lower incisors, in a case where both the primary canine and lateral incisor were missing in one quadrant (Fig. 3).

**Table 2:** Distribution of the missing primary teeth due to *Ebiino* among the study participants

Tooth # (%)									
55	54	53	52	51	61	62	63	64	65
0	2 (0%)	25 (0.3%)	3 (0.0%)	2 (0.0%)	0	3 (0.0%)	24 (0.3%)	2 (0.0%)	0
85	84	83	82	81	71	72	73	74	75
0	1 (0.0%)	29 (0.3%)	2 (0.0%)	0	0	0	27 (0.3%)	3 (0.0%)	0

*n* = 8640 (total number of teeth of the participants)

## Discussion

During the study of the 432 participants, a record of missing teeth due to reasons other than caries and trauma was made. The pattern of the missing primary canines was rather unique, hence the interest to learn reasons for their absence. The absence was attributed to a traditional practice called *Ebiino*, a practice that appears to be rife in this region and different regions of Uganda [2, 8]. In the current study, only visual observations of the teeth were made, and no radiographs or other investigative methods were applied. This combined with the fact that some children could not be examined due to either being absent at school on the day of examination, were disruptive and hence not examined or lacked consent from their caregivers to participate in the study, could have had some effects on the results obtained.

Nonetheless, the prevalence of *Ebiino* in the study population was 8.1%, which was lower than what was reported before in different regions and age groups in Uganda. Bataringaya et al. [2] reported a prevalence of IOM of 28% due to missing permanent canines among 14 year old children in Kampala, while in 1969, a prevalence of IOM of 16% of members of the Acholi community from Northern Uganda. A study by Tirwomwe et al. [8] reported a 28.3% prevalence of traditional birth extractions (TBE) among children aged 36 months and below in the surveyed Ugandan districts of Arua (North-western), Gulu (Northern), Kabale (South-western), Kampala (Central), Masindi (Western) and Mbale (Eastern). The prevalence of TBE in the study was highest in Gulu district (55.1%), followed by Arua (41.0%) and Masindi (36.1%). The rest of the districts of Mbale, Kabale and Kampala had prevalence of 22.5, 21.8 and 17.6% respectively. In these studies, the primary canine was found to be the most affected tooth and both jaws were equally affected like in the current study. These values are relatively higher when related to those found in the current study, especially when related to those for Kabale district that neighbours Rukungiri district. This could be attributed to the health education that has been carried out through out the country against the practice. Further, the sampling technique used and the age considered could have influenced the prevalence rate reported in earlier studies. Of note also is the fact that when the outcomes of the Ugandan studies already done are analysed and compared, the Northern region of the country appears to be more affected with IOM than the southern region. Furthermore, the widespread occurrence of *Ebiino* in Uganda could suggest that more communities still believe in the myth that the extraction of primary canine tooth buds prevents or treats childhood illnesses.

Higher prevalence figures of IOM have also been reported in other East Africa region, with reported prevalence for example Kenya of 72–87% among Maasai children in 1995) [9], Tanzania of 5.2–16.9% according to a 2015 review [4], Ethiopia of 15% for primary canines and 7% for damaged permanent canines as a result, in 2013 report [10].

There are also studies in the developed/other developing countries that have reported high prevalence rates among immigrants from Africa. In Israel for example, in a study conducted in 2013, the prevalence of missing primary canines or canines with dental anomalies among children of immigrant Ethiopians was 60%. This prevalence for Israel was higher when compared to 12.5 and 7.4% of younger and older native Israeli children respectively as reported in that study [6]. In many of the regions where *Ebiino* has been reported, communities with a low socio-economic status populate these regions.

The practice of *Ebiino* affects the child’s health and well being, in spite of the myth held by those who undertake the procedure, that IOM would prevent or treat childhood diseases and prevent death. In fact, the immediate, short- and long-term, local and systemic complications have been observed [1, 4], There is the excessive bleeding associated with IOM, which can predispose the affected children to anaemia. The use of poorly sterilised instruments or tools to perform the invasive procedure predisposes the child to septicaemia and heightens the risk of contracting infectious diseases, such as HIV, Hepatitis B and Tetanus [1, 3]. The crude methods used accompanied by the associated trauma and pain for the infant qualifies it to be a form of child abuse. Case reports of the occurrence of a radicular cystic lesion [11] and noma [12] have been reported to occur in infants following this practice. Death also occurs, with a 21% fatality rate reported in a Ugandan study [2].

In the present study, besides the missing primary canines, there were also other teeth missing that included the primary lateral incisors and primary first molars. This could have resulted from trauma during the enucleation of the primary canines. The early loss of these teeth can affect function (mastication and speech) and can also lead to drifting of adjacent teeth into the edentulous space, reduction in arch length ultimately affecting the occlusion [13]. Also found in the study was damage to the adjacent teeth in the form of hypoplastic lesions on some of the primary lateral incisors and primary first molar. In some cases, primary canines that had not been successfully removed were found to be damaged or hypoplastic (Figs. 3 and 4).

Damage to the tooth buds of the permanent successors has been reported and usually results in missing or defective permanent teeth or may affect their eruption time [2, 14]. A study by Hassanali et al., also noted a significant reduction in the arch size among Maasai children whose mandibular permanent central incisors had been traditionally extracted, in another form of IOM/ritual practiced in this community [15]. All these effects of *Ebiino* impacts negatively on the occlusal traits in the permanent dentition and contributes to the development of malocclusions, which would be costly to manage [2].

## Conclusion

The prevalence rate of *Ebiino* (IOM), involving the gauging of primary canines, was 8.1% in the current study, indicating that *Ebiino* is still prevalent in Nyakagyeme sub-county, Rukungiri district, Uganda. The practice has associated negative effects to the developing dentition in the child in the form of early loss of adjacent teeth, dental trauma, hypoplasia, midline shift and dental displacement. It would be suffice to further state that IOM should be considered as a form of child abuse that requires to be eradicated from the concerned communities.

## Data Availability

The data that provide the basis for the presented results of this study is available by contact to the corresponding author, but restrictions apply to the availability of these data and to a certain time period, as the data were used under license for the current study, and so are not publicly available.

## Abbreviations

Intra-ACP: Intra Africa Carribean Pacific
IOM: Infant Oral Mutilation
PDWG: Paediatric Dentistry Working Group
PI: Principal Investigator
SD: Standard deviation
WHO: World Health Organization
